# Curcumin combined with FAPαc vaccine elicits effective antitumor response by targeting indolamine-2,3-dioxygenase and inhibiting EMT induced by TNF-α in melanoma

**DOI:** 10.18632/oncotarget.4577

**Published:** 2015-07-20

**Authors:** Guan-Min Jiang, Wan-Ying Xie, Hong-Sheng Wang, Jun Du, Bai-Ping Wu, Wei Xu, Hui-Fang Liu, Ping Xiao, Zhi-Gang Liu, Hong-Yan Li, Shuang-Quan Liu, Wen-Jun Yin, Qiu-Gui Zhang, Jian-Ping Liang, Hong-Jun Huang

**Affiliations:** ^1^ Department of Clinical Laboratory, Hunan Cancer Hospital & The Affiliated Cancer Hospital of Xiangya School of Medicine, Central South University, Changsha, 410013, PR China; ^2^ Department of Clinical Laboratory, The First Affiliated Hospital of University of South China, Hengyang, 421001, PR China; ^3^ Department of Microbial and Biochemical Pharmacy, School of Pharmaceutical Sciences, Sun Yat-sen University, Guangzhou, 510006, PR China; ^4^ Department of Radiation Oncology, Hunan Cancer Hospital & The Affiliated Cancer Hospital of Xiangya School of Medicine, Central South University, Changsha, 410013, PR China; ^5^ Department of Thoracic Surgery 2, Hunan Cancer Hospital & The Affiliated Cancer Hospital of Xiangya School of Medicine, Central South University, Changsha, 410013, PR China

**Keywords:** curcumin, indolamine-2,3-dioxygenase, immunotherapy, melanoma, fibroblast activation protein α

## Abstract

Fibroblast activation protein α (FAPα) is a potential target for cancer therapy. However, elimination of FAPα+ fibroblasts activates secretion of IFN-γ and TNF-α. IFN-γ can in turn induce expression indolamine-2,3-dioxygenase (IDO), thereby contributing to immunosuppression, while TNF-α can induce EMT. These two reactive effects would limit the efficacy of a tumor vaccine. We found that curcumin can inhibit IDO expression and TNF-α-induced EMT. Moreover, FAPαc vaccine and CpG combined with curcumin lavage inhibited tumor growth and prolonged the survival of mice implanted with melanoma cells. The combination of FAPαc vaccine, CpG and curcumin stimulated FAPα antibody production and CD8+ T cell-mediated killing of FAPα-expressing stromal cells without adverse reactive effects. We suggest a combination of curcumin and FAPαc vaccine for melanoma therapy.

## INTRODUCTION

Melanoma is the most serious form of skin cancer. Historically, metastatic melanoma has carried a poor prognosis, but recently new and potentially more effective anti-CTLA4 and anti-PD-1 therapies, as well as other immunotherapies, have shown promise [1, 2]. However, a major impediment to cancer immunotherapy is the induction of indolamine-2,3-dioxygenase (IDO), which is a key mediator of tumor immune tolerance [3]. IDO is the first and rate-limiting enzyme in the metabolism of the essential amino acid tryptophan along the kynurenine pathway. It induces immune tolerance by depleting tryptophan locally and producing toxic tryptophan catabolites that induce proliferation arrest, inactivation and apoptosis among T lymphocytes and natural killer (NK) cells [4, 5]. Thus, inhibition of IDO expression and/or activity is considered to be a potentially effective therapeutic strategy [6, 7].

Given the close interaction between tumor cells and cancer-associated fibroblasts (CAFs) in the tumor microenvironment, CAF-targeted strategies could also make an important contribution to an integrated cancer immunotherapy [8]. Fibroblast activation protein α (FAPα) is a serine protease involved in extracellular matrix remodeling and is strongly expressed by CAFs, but is not detectable in normal adult human tissues [9, 10]. Furthermore, because of the multiple roles played by FAPα in neoangiogenesis, invasion and metastasis, it is frequently explored as a target for cancer therapy. In the context of immunotherapy involving T cells targeting cancer cells, an agent targeting FAPα-expressing cells might increase therapeutic efficacy against both solid tumors and metastatic cells [11, 12]. Removing FAPα+ fibroblasts does not alter the number or subtypes of tumor infiltrating T cells, but does result in their activation and secretion of interferon-γ (IFN-γ) and tumor necrosis factor-α (TNF-α), two antitumor cytokines secreted by activated Th1 and NK cells [13]. On the other hand, IFN-γ also strongly induces expression of IDO, which reportedly contributes to tumor-induced immunosuppression [14], while TNF-α can induce epithelial-mesenchymal transition (EMT), an important contributor to cancer invasion and metastasis [15–18]. Together, the induction of IDO expression and EMT limit the therapeutic efficacy of antitumor vaccines.

Curcumin is a natural polyphenolic compound derived from the root of curcuma longa, which is widely used for medical purposes in India. A large body of evidence from *in vitro* and *in vivo* studies in both animals and human indicate that curcumin exerts antioxidant, anti-inflammatory, antitumor, antifibrotic and wound healing properties [19, 20]. Moreover, we previously showed that curcumin can inhibit the IDO expression induced by IFN-γ and the EMT induced by TNF-α, suggesting curcumin could be a useful agent for antitumor therapy when combined with tumor vaccines.

In our present work, we assessed the therapeutic efficacy and molecular mechanism of a combination of curcumin with FAPαc vaccine, which contains the main catalytic domain of the dipeptidyl peptidase of FAPα with abundant T-cell and B-cell epitopes. In addition, we also administered the Th1-polarized immunoregulator CpG oligonucleotide 1826, which was previously shown to enhance specific cellular and humoral immune responses in C57 mice [21]. It was anticipated that this new approach would eliminate the immune tolerance and tumor cell metastasis induced by IFN-γ and TNF-α. Our results show that FAPαc vaccine in combination with curcumin inhibits tumor growth and significantly prolongs the survival of mice implanted with B16 melanoma cells. These findings suggest combination therapy using FAPαc vaccine in combination with curcumin may be an effective approach to preventing and treating melanoma.

## RESULTS

### Curcumin dose-dependently down-regulates IDO expression in B16 melanoma cells

To investigate the effect of curcumin on IDO expression, B16 melanoma cells were first pretreated for 4 h with selected concentrations of curcumin before treating the cells with IFN-γ (100 U/ml) for 24 h. IDO expression was then assessed in total cell lysates using western blotting. As shown in Figure 2, treatment with curcumin significantly and dose-dependently reduced IDO expression, which was almost completely inhibited at a concentration of 25 μM (Figure 2). More importantly, curcumin not only inhibited IDO expression induced by IFN-γ, it also suppressed basal expression of IDO in B16 cells. But because the majority of B16 exposed to 25 μM curcumin died, in subsequent experiments we applied curcumin at a concentration of 15 μM, which downregulated IDO expression significantly but did not affect the survival rate of the cells (Figure 2A–2B). Overall, this result demonstrates that curcumin down-regulates IFN-γ-induced and basal expression of IDO in a dose-dependent manner.

**Figure 1:**
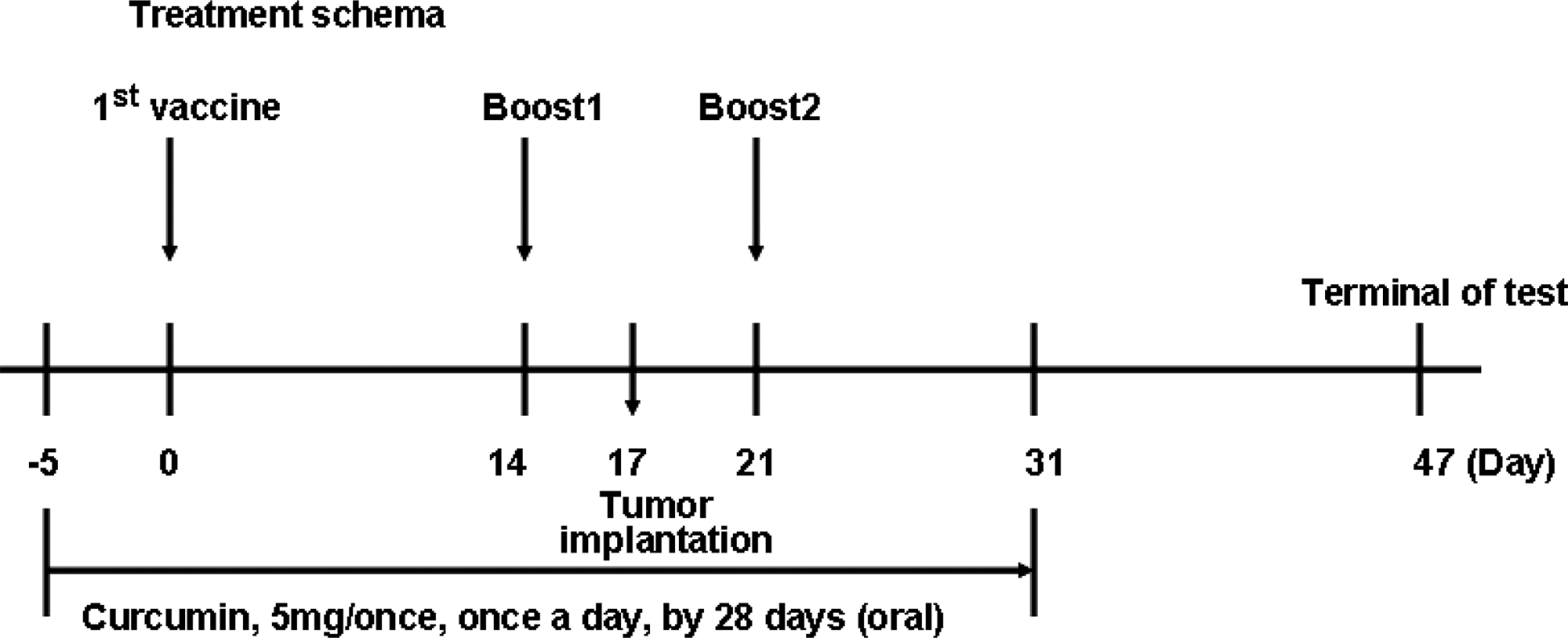
Schematic representation of the treatment schedule and dosages in mice

**Figure 2:**
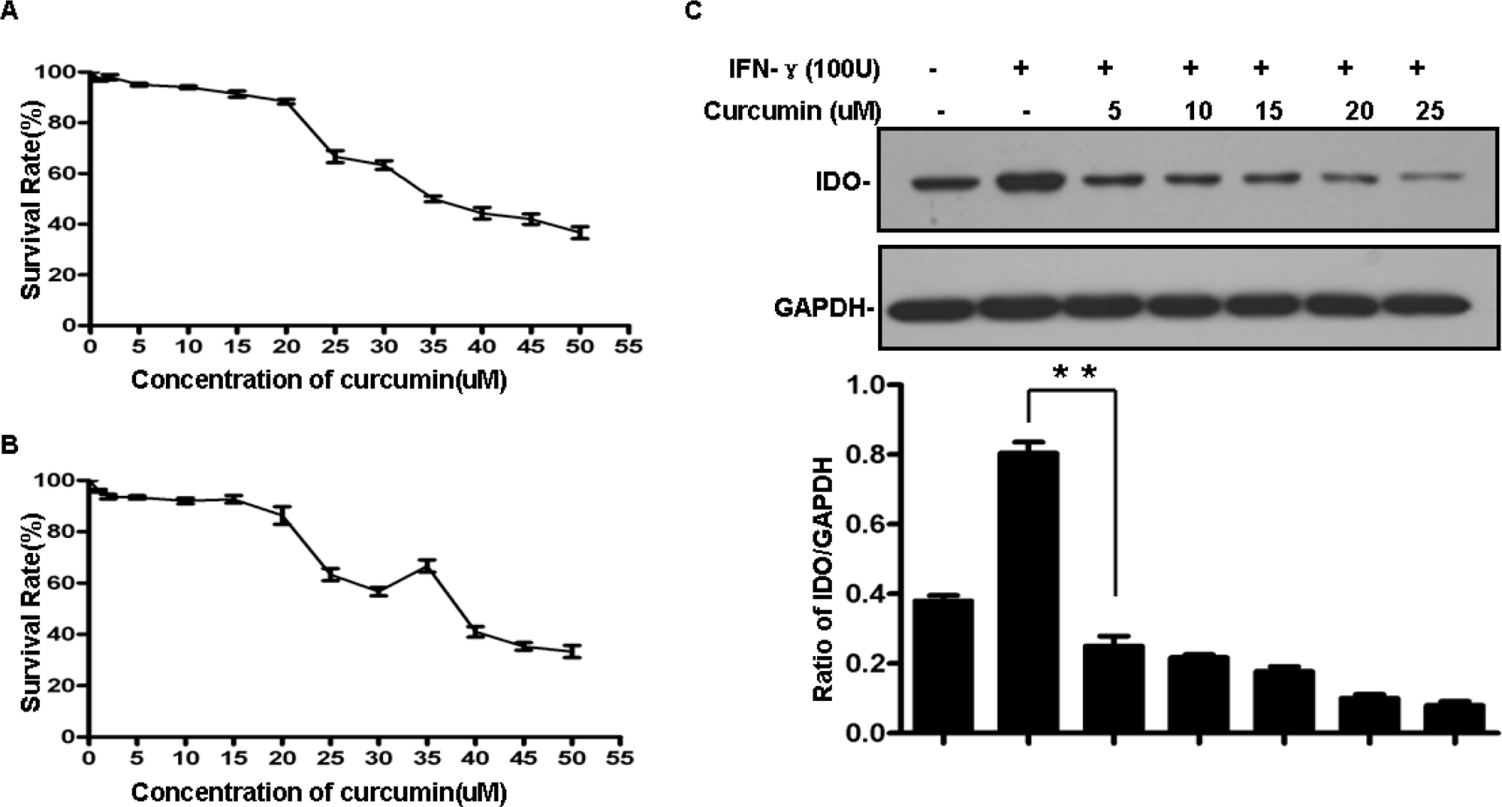
**A, B. Cytotoxicity of curcumin in B16 cells.** Experiments were performed by means of an MTT enzyme assay. B16 cells were incubated in the presence of different concentrations of curcumin at 37°C for 24 h (A) or 48 h (B) Each column represents the mean ± SD with respect to 100% control. At least three independent assays were performed. **C.** Curcumin down-regulated the IFN-γ-induced expression of IDO in a dose-dependent manner. B16 cells were pretreated with the indicated concentrations of curcumin for 2 h and then treated with 100 U/ml IFN-γ for 24 h. IDO expression was detected by western blotting, GAPDH served as the loading control. Similar results were obtained in three independent experiments.

### Curcumin suppresses TNF-α-induced EMT in B16 melanoma cells

**Figure 3:**
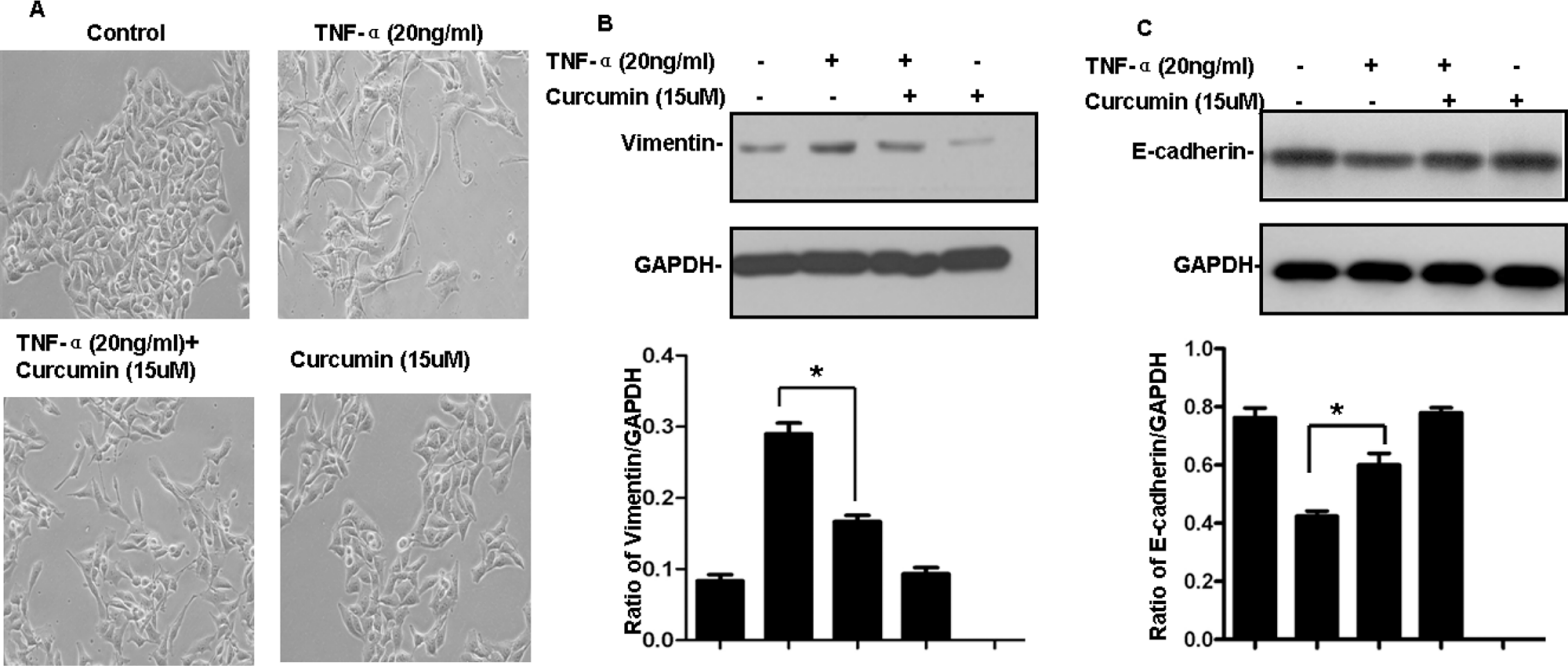
Curcumin inhibits TNF-α-induced EMT in B16 cells **A.** Cells were pre-treated with or without 15 μM curcumin for 4 h and then treated with 20 ng/ml TNF-α for 72 h. Phenotypic changes of EMT in B16 cells were detected using a phase contrast microscope. **B, C.** B16 cells were treated as indicated in (A) after which expression of vimentin (B) and E-cadherin (C) was detected by western blotting. GAPDH served as the loading control. Similar results were obtained in three independent experiments, *p < 0.05.

As mentioned, TNF-α has been shown to induce EMT in several cell lines. To verify that effect in B16 cells, we treated the cells with TNF-α (20 ng/ml) and recorded the phenotypic changes using a phase contrast microscope. After treatment with TNF-α for 72 h, B16 cells became scattered and adopted the typical fibroblast-like morphology of mesenchymal cells (Figure 3A). In addition, up-regulation of vimentin and down-regulation of E-cadherin are important molecular markers of EMT. TNF-α-treated cells in the present study exhibited significantly up-regulated vimentin expression and down-regulated E-cadherin expression (Figure 3B–3C). Taken together, these results indicate that TNF-α induces EMT in B16 cells.

To then evaluate the effect of curcumin on TNF-α-induced EMT, the cells were pretreated for 4 h with or without curcumin (15 μM) before treatment for 72 h with or without TNF-α (20 ng/ml). Phenotypic changes were then recorded using a phase contrast microscope, and vimentin and E-cadherin expression was assessed using western blotting. We found that in the absence of curcumin TNF-α-treated cells exhibited morphological changes characteristic of EMT as well as a significant increase in vimentin levels and a corresponding decrease in E-cadherin levels. By contrast, the morphological changes induced by TNF-α were not observed in cells pretreated with curcumin, and the vimentin and E-cadherin levels were similar to those in the control group (Figure 3B–3C). This suggests that curcumin suppresses TNF-α-induced EMT in B16 melanoma cells.

### IDO and FAPα were detectable in B16 tumor-bearing mice

**Figure 4:**
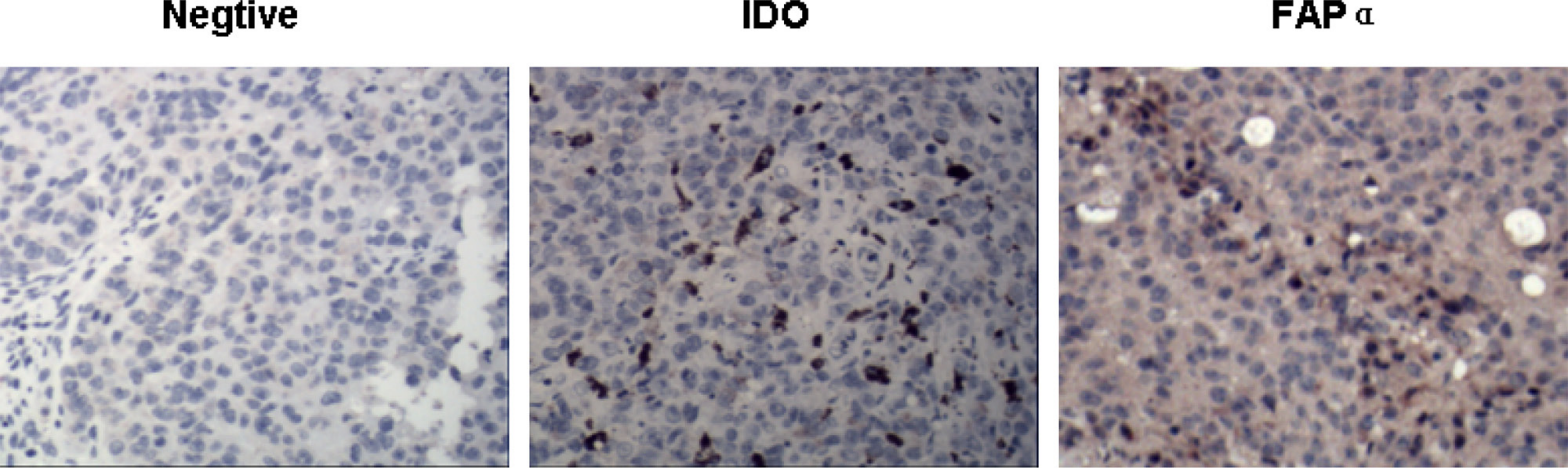
Identification of FAP α and IDO expression in B16 tumor cells of mice Distributions of IDO and FAP α in B16 tumor tissue was examined immunohistochemically (400×). IDO was observed in regions of B16 tumor cells, while FAP α was mainly detected in tumor stroma cells.

Because IDO is believed to be an important mediator of tumor immune tolerance that creates an immunosuppressive microenvironment within the tumor site, while the tumor-associated antigen FAPα plays multiple roles in neoangiogenesis, invasion and metastasis, we established an IDO- and FAPα-expressing tumor model by implanting B16 cells into female C57 mice. We then performed immunohistochemical studies to determine whether IDO and FAPα were, in fact, expressed by the tumor tissues. As shown in Figure 4, we detected IDO expression in regions of B16 tumor tissue and FAPα expression in fibroblasts within the tumor stroma. These findings suggest that IDO-mediated immune tolerance occurs in B16 tumor-bearing mice, and FAPα is a useful target for tumor immunotherapy.

### FAPαc vaccine and CpG combined with curcumin lavage elicits a protective antitumor response in a FAPα-positive mice model

**Figure 5:**
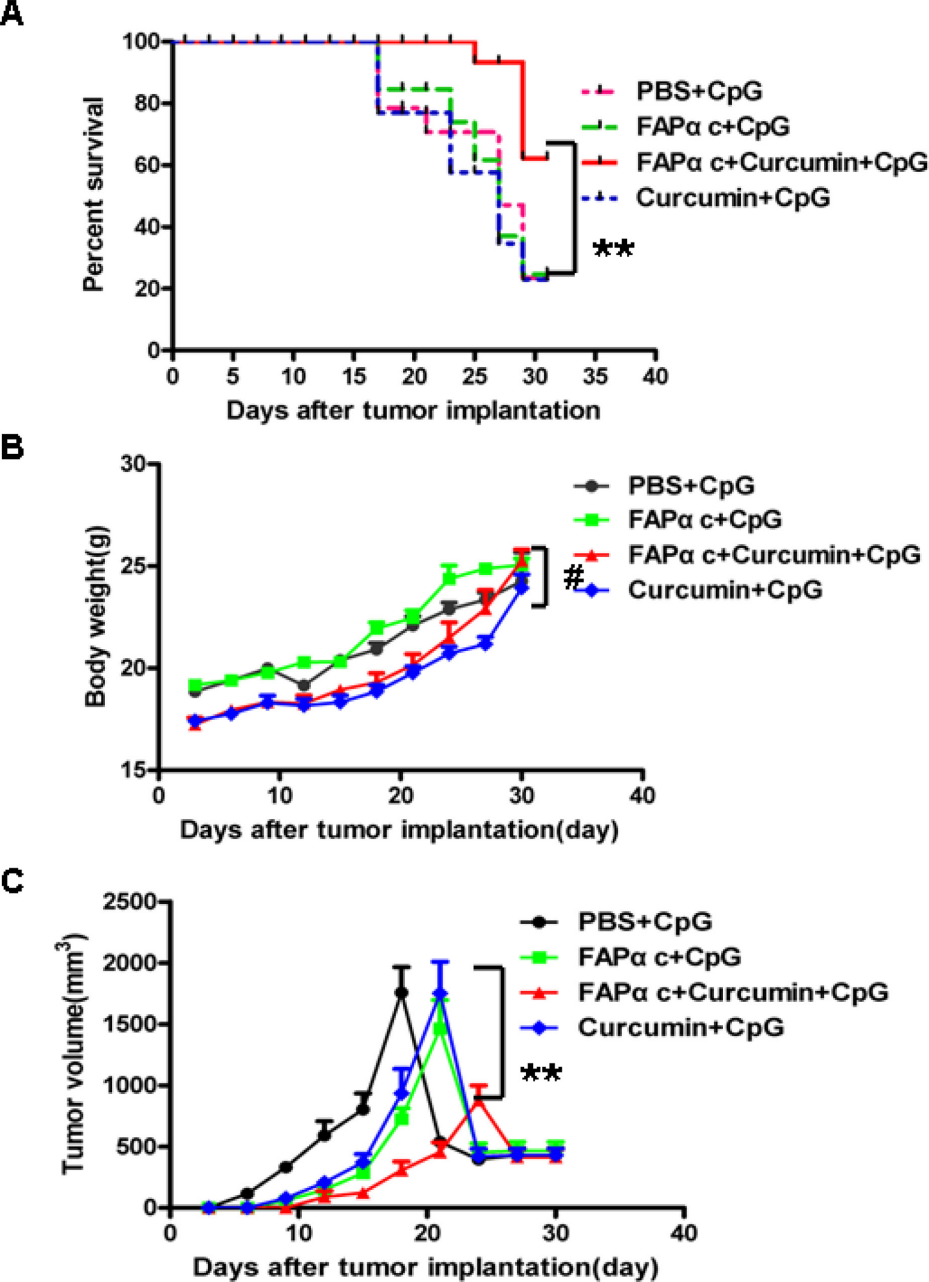
FAPαc vaccine plus curcumin and CpG elicited a protective antitumor response in B16 tumor-bearing mice B16 tumor cells were implanted (s.c.) in the backs of B57 mice after the second immunization, after which tumor volumes and body weights were measured at regular intervals. **A.** Survival rate among mice treated during the experiment. Thirty-one days after tumor implantation, the survival rate among mice immunized with FAPαc+curcumin+CpG is significantly higher than among mice immunized with PBS+CpG, FAPαc+CpG or curcumin+CpG (**p < 0.01). **B.** Average body weights of the mice in the different groups did not differ during the experiment (#*p* > 0.05). **C.** Tumor growth (*n* = 10). Following vaccination, tumors were measured every 3 days (mean ± SE) as described in the Materials and Methods. Tumor volume in mice immunized with FAPαc+curcumin+CpG were significantly smaller than in the other three groups (**p < 0.01).

We demonstrated *in vitro* that curcumin down-regulates IDO expression and suppresses TNF-α-induced EMT (Figures 2 and 3). To assess the preventive efficacy of FAPαc vaccine combined with curcumin *in vivo*, experiments were performed using a FAPα-positive B16 tumor model. We found that 31 days after tumor implantation, the survival rate among mice immunized with FAPαc vaccine and CpG combined with curcumin lavage was 60%, whereas the survival rates among mice immunized with CpG alone, with FAPαc plus CpG or with curcumin plus CpG were all 20% (Figure 5A). This suggests FAPαc vaccine and CpG in combination with curcumin lavage elicits a protective antitumor response in FAPα-positive B16 tumor-bearing mice. Consistent with that idea, the B16 tumor volume in mice treated with FAPαc vaccine and CpG combined with curcumin lavage was significantly smaller than in the other three groups 21 days after tumor implantation. During the period from 21 days to 31 days after tumor implantation, tumor volumes decreased sharply in all four groups, due to the development of ulcers (Figure 5C). In addition, the body weights of the mice in all four groups continued to increase, but there were no significant differences among the four groups (Figure 5B). Overall, these results suggest that the antitumor activity of the combination of FAPαc vaccine and CpG with curcumin lavage was significantly greater than CpG alone, curcumin plus CpG or even FAPαc vaccine plus CpG.

### Antitumor mechanism of the FAPαc vaccine combined with curcumin

**Figure 6:**
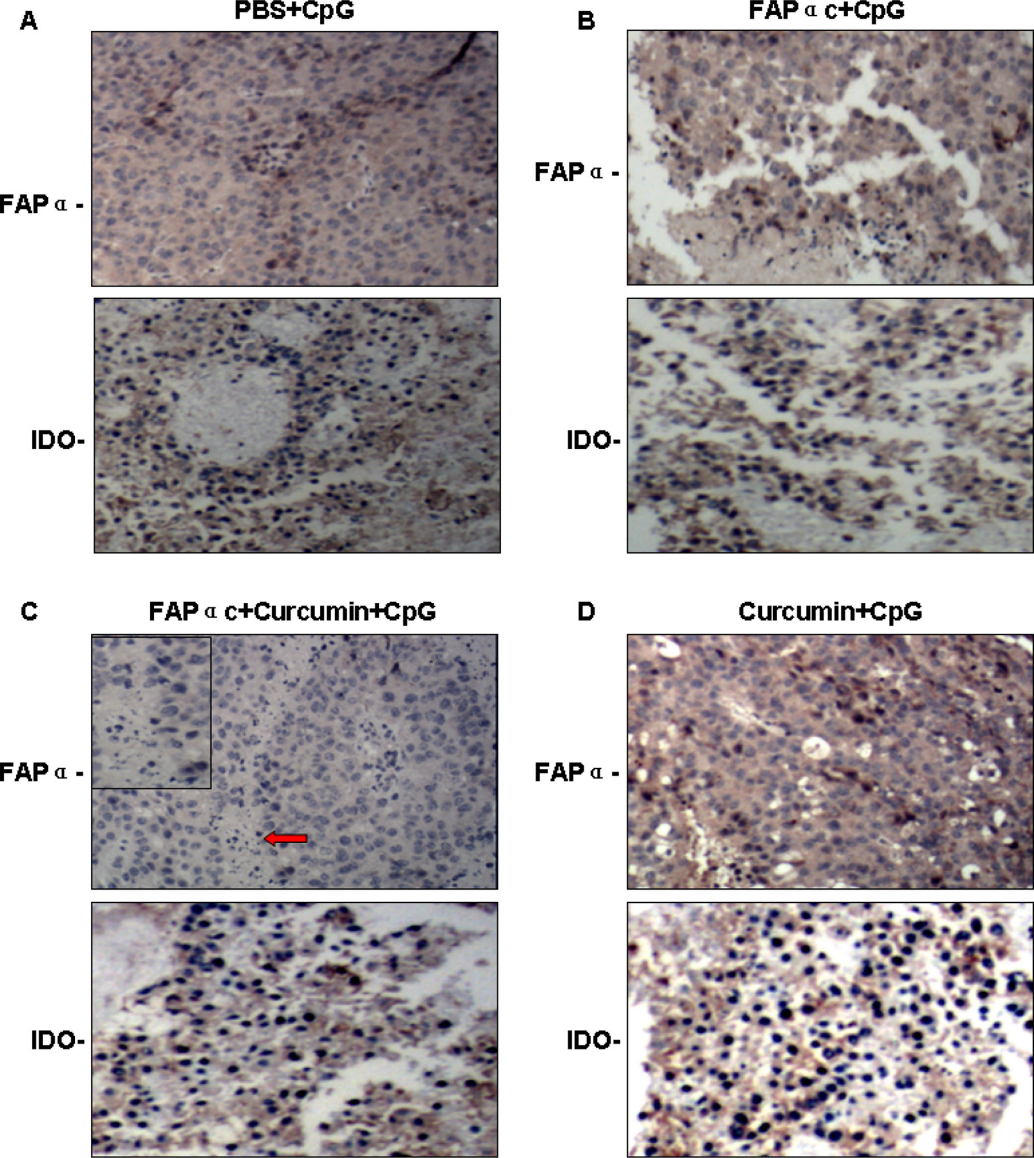
Effect of curcumin and FAPαc vaccine on expression of FAPα and IDO in B16 tumor-bearing mice Mice were assigned to four groups as follows, PBS+CpG **A.** FAPαc+CpG **B.** FAPαc+curcumin+CpG **C.** Curcumin+CpG **D.** after which the distributions of IDO and FAPα in tumor tissue were examined immunohistochemically.

**Figure 7:**
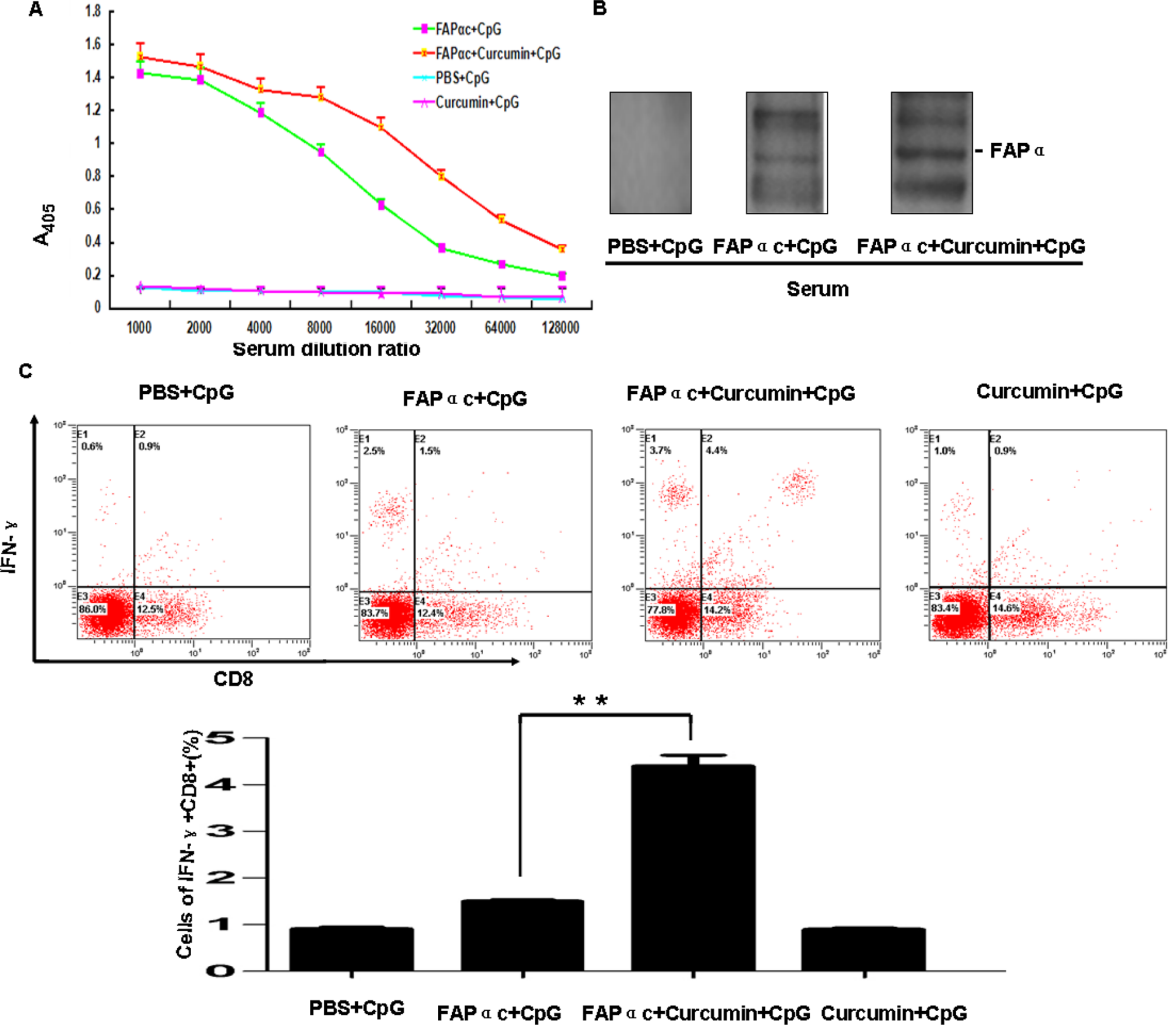
Detection of immune responses in B16 tumor-bearing mice **A.** Results of ELISAs using an anti-FAPα antibody. Mice were vaccinated as described in Materials and Methods. One week after the second booster, the serum samples were collected for detection of anti-FAPα antibody using the ELISA. **B.** Western blotting analysis of the serum anti-FAPα antibody specificity. The antigen was the lysate from tumor tissue from B16 tumor-bearing mice. **C.** Detection of CD8+ T cell function. On day 47, when the experiment was terminated, splenocytes were isolated and re-stimulated with FAPαc *ex vivo*. IFN-γ production in CD8+ T cells was then assessed using flow cytometry, **p < 0.01.

To investigate the antitumor mechanism of FAPαc vaccine combined with curcumin, we first performed immunohistochemical studies to verify the expression of IDO and FAPα within the tumor tissues. As shown in Figure 6, we found that in mice immunized with FAPαc vaccine and CpG combined with curcumin lavage, the numbers of tumor cells expressing IDO and FAPα were significantly reduced and the tumor stroma was obviously destroyed (Figure 6C, red arrow). This indicates that curcumin abrogated the immune tolerance, most likely by suppressing IDO expression, enabling the FAPαc vaccine to stimulate an important antitumor immune response *in vivo*. As shown in Figure 7A, mice immunized with FAPαc vaccine and CpG in combination with curcumin lavage exhibited a more significant humoral immune response than mice immunized with FAPαc vaccine and CpG without curcumin (each group, *n* = 10; *P* < 0.01), though both groups elicited greater humoral immune responses than mice immunized with CpG alone or with curcumin plus CpG (each group, *n* = 10; *P* < 0.01).

This specific immune response was examined further using western blotting, which showed that the anti-FAPα antibody from individual immunized mice recognized FAPα protein in lysates of tumor tissue from B16 tumor-bearing mice. By contrast, no bands appeared when using the serum from mice immunized with PBS and CpG (Figure 7B). These data indicate that the FAPαc vaccine induces an antibody specific to the FAPα antigen.

To further explore the antitumor mechanism of FAPαc vaccine combined with curcumin, splenic CD8+ T cell function was examined. Splenocytes isolated from immunized mice were restimulated with FAPαc for 3 days, after which IFN-γ production by the CD8+ T cells was assessed using flow cytometry. As shown in Figure 7, we found that nearly 4.4% of the CD8+ T cells produced IFN-γ in mice immunized with the FAPαc vaccine and CpG combined with curcumin lavage. By contrast, only 0.9% of CD8+ T cells in mice immunized with CpG alone, 0.9% in mice immunized with CpG and curcumin lavage, and 1.5% in mice immunized with FAPαc and CpG produced IFN-γ. These results suggest that the FAPαc vaccine and CpG combined with curcumin lavage elicited a protective antitumor response that was partially dependent on a curcumin-induced increase in FAPα antibody and CD8+ T cell-mediated killing of FAPα-expressing tumor stromal cells.

## DISCUSSION

Immunotherapy is poised to assume a more central role in the treatment of a variety of cancer types. Recognition of the immunogenic nature of melanoma has resulted in the development of several immunotherapeutic agents for its treatment, including ipilimumab and pembrolizumab, antibodies respectively blocking CTLA-4 and PD-1 [22]. However, antigen selection remains a crucial step in tumor immunotherapy. It was recently suggested that tumor stromal cells could be a useful target for tumor immunotherapy because these cells, unlike the tumor cells, are diploid, genetically stable and susceptible to immunological attack [23].

Stromal cells and cancer cells depend on one another for mutual paracrine stimulation, and stromal CAFs are likely required for the survival and growth of cancer cells [24]. The transmembrane serine protease FAPα is highly expressed on the CAFs present in >90% of human epithelial tumors, and plays a significant role in tumor progression and metastasis [25]. Targeting FAPα genetically or with vaccines or pharmacological agents has been shown to impair tumor progression in several preclinical cancer models [26, 27]. Consequently, FAPα is thought to be an adaptive tumor-associated antigen useful for tumor immunotherapy. In our research, we found that FAPα is stably expressed by the tumor stromal cells in B16 melanoma-bearing mice (Figure 4). The FAPαc peptide used in the present study is the main catalytic domain of the dipeptidyl peptidase of murine FAPα and contains abundant T cell and B cell epitopes, and is 96% homologous to the human FAPα homolog [28]. This suggests a FAPαc vaccine could prove to be a valuable reference for clinical trials.

Although strategies targeting the immune system to achieve an antitumor response have shown promise in terms of survival benefit, the most common challenges encountered during immune-based antitumor therapy are lack of an antitumor immune response and frequent adverse events [29, 30]. For example, as mentioned above, the secretion of the antitumor cytokines IFN-γ and TNF-α by activated Th1 and NK cells is a feature of effective tumor vaccines. Unfortunately, however, IFN-γ can also induce IDO expression and TNF-α can induce EMT, limiting the development of a beneficial effect of therapy [13–15]. Here we found that these two adverse events also occur in B16 melanoma cells, and that these cells express IDO under basal conditions (Figures 2 and 3) and in the tumor tissue of B16 melanoma-bearing mice (Figure 4). This implies that IDO mediates immune tolerance in B16 tumor-bearing mice and suggests, therefore, that inhibiting induction of IDO expression and EMT could improve the efficacy of tumor vaccines. Notably, we found that curcumin inhibits both basal and IFN-γ-induced IDO expression and simultaneously represses TNF-α-induced EMT in B16 melanoma cells (Figures 2 and 3). Curcumin reportedly suppresses IFN-γ-induced IDO expression by blocking the Janus-activated kinase/protein kinase Cδ/STAT1 signaling pathway [31]. In addition, it also inhibits lipopolysaccharide-induced EMT in breast cancer cells through down-regulation of NF-κB/Snail signaling [32]. Given that TNF-α induces EMT and promotes metastasis via NF-κB-mediated Twist expression [33], we speculate that curcumin may inhibit TNF-α-induced EMT in B16 cells by inhibiting NF-kB-dependent signaling, though that idea remains to be tested. The properties of curcumin appear to make it an ideal agent for combination with tumor vaccines for antitumor therapy. In addition, we previously observed that adding CpG to the vaccine dramatically enhances the vaccine's antitumor efficacy [21]. Taken together, these results suggest combining FAPαc, curcumin and CpG could be a potentially effective approach to treating melanoma (Figure 5).

In sum, our findings suggest a FAPαc vaccine in combination with curcumin is a promising therapeutic strategy for the treatment of melanoma. This approach is associated with three beneficial effects: First, it breaks tumor tolerance and activates tumor-responsive T cells through inhibition of IDO expression. Second, because cancer cells depend on stromal cells for support through paracrine stimulation, targeting stromal cells may increase the susceptibility of melanoma cells to both immunological and pharmacological antitumor therapy (Figure 6). Finally, curcumin inhibits TNF-α-induced EMT, thereby suppressing the invasiveness and metastatic potential of melanoma cells. We therefore conclude that combined administration of FAPαc vaccine with curcumin may be a new and effective method for preventing and/or treating melanoma.

## MATERIALS AND METHODS

### Chemicals and reagents

Curcumin, hematoxylin, Freund's incomplete adjuvant, mouse TNF-α and IFN-γ were purchased from Sigma-Aldrich (Deisenhofen, Germany). The monoclonal FAPα antibody was from Abcam, and the monoclonal IDO antibody was from Chemicon. The monoclonal anti-vimentin antibody, polyclonal anti-β-actin and secondary HRP-conjugated anti-mouse antibody were from Cell Signaling Technology (MA, USA). The anti-CD8-PE and anti-IFN-γ-FITC were from Beckman Coulter, and IL-2 and Brefeldin A were from BioLegend.

### Cell line and mouse

B16 (ATCC^®^ CRL-6475™) cells were cultured in RPMI 1640 (Gibco, Germany) containing 10% fetal calf serum (Gibco, Germany). C57 mice were purchased from the Sun Yat-sen University (Guangzhou, China) animal center and raised under pathogen-free conditions. All animal studies were conducted in accordance with institutional guidelines for the care and use of experimental animals.

### Preparation of FAPac

We chose the main catalytic domain of the dipeptidyl peptidase of murine FAPα (AA599-AA696) (NP_032012.1), which contains abundant T-cell and B-cell epitopes. The target gene (mRNA 1967–2258, GenBank: BC019190.1) was obtained by RT-PCR and ligated with a prokaryotic expression vector, pET28a(+) (Novagen, Germany), which was then amplified in *E. Coli* DH5α and expressed in *E. coli* BL21 (DE3). The purified peptide (FAPαc) was employed as the FAPα vaccine.

### Cytotoxicity assay

The cytotoxicity of curcumin toward the cultured cells was assessed using MTT [3-(4, 5-dimethylthiazol-2yl)-2, 5-diphenyltetrazolium bromide] assays (Sigma Chemical Co., USA). B16 cells were seeded onto 96-well microplates (NunC, Denmark) at a density of 1 × 10^4^ cells per well and incubated for 24 h. The Cells were then treated with selected concentrations of curcumin for 24 h or 48 h. Cells in culture medium alone served as the untreated control. The MTT reagent (5 mg/ml in distilled water) was prepared immediately prior to use. After removing the incubation medium from the wells, the cells were washed with phosphate-buffered saline (PBS) and 10 ml of MTT reagent was added. After incubation for 4 h at 37°C, MTT reagent in 100 ml of dimethylsulfoxide (DMSO) was added to each well. Surviving cells were then detected by measuring absorbance at 570 nm using a plate reader. The cell viability was expressed as a percentage of the values obtained for the controls.

### Western blotting analysis

Cells were lysed in lysis buffer, after which the lysates were cleared by centrifugation and denatured by boiling in Laemmli buffer. Aliquots of protein samples were then separated on 12% sodium dodecyl sulfate (SDS)-polyacrylamide gels and electrophoretically transferred to nitrocellulose membranes. After blocking with 5% non-fat milk at room temperature for 2 h, the membranes were incubated first with the primary antibody at 1:1000 dilution overnight at 4°C, and then with a HRP-conjugated secondary antibody at 1:5000 dilution for 1 h at room temperature. Specific immune complexes were detected using western blotting plus chemiluminescence reagent (Life Science, Inc., Boston, MA).

### Adjuvant and vaccine preparation

The phosphorothioate-modified CpG oligonucleotide 1,826 (5′-TCCATGACGTTCCTGACGTT-3′) was synthesized by the Shanghai Sangon Biological Engineering Technology and Service. The antigens FAPαc and CpG were separately dissolved in PBS. The two solutions were mixed to obtain a suspension including 200 μg (first vaccine per mouse) or 100 μg antigens FAPαc and 30 μg CpG. Then the suspension was emulsified by equivalent volume of Freund's incomplete adjuvant.

### Immunization and measurement of tumor growth

Five-week-old female C57 mice were randomly assigned into four groups, and immunized (s.c.) on the back in multiple locations on days 0, 14 and 21 (Figure 1). The groupings were as follows: 1 (*n* = 10), 30 μg CpG (per mouse); 2 (*n* = 10), 200 μg FAPαc and 30 μg CpG; 3 (*n* = 10), 200 μg FAPαc, 30 μg CpG and curcumin (administration was initiated with the first immunization and ended 10 days after the last immunization; dissolved in 1% Tween80/0.5% methylcellulose at a concentration of 50 mg/ml, 5 mg once/day) with additional lavage using intragastric administration needles (0.9 × L50 mm); 4 (*n* = 10), curcumin and 30 μg CpG. On day 17, tumors were implanted through injection of B16 cells (10^5^ cells) into the backs of the mice. Tumor growth and body weights were measured every three days, and the tumor volumes were determined using the formula (a × b^2^)/2, where a is the larger and b the smaller diameter. The experiment was terminated on day 47.

### Immunohistochemical detection of FAPα and IDO expression

Tumors were harvested and then fixed in 4% paraformaldehyde, after which 5-μm paraffin-embedded sections were cut. This was followed by deparaffinization, antigen retrieval, serum blocking and incubation with anti-FAPα antibody (1:100 dilution) or anti-IDO antibody (1:50 dilution) overnight at 4°C. An isotype-matched irrelevant antibody was used as the negative control. After washing, slides were incubated with HRP IgG (EnVision+; DAKO), and DAB (DAKO) was applied as a substrate. Lastly, the slides were counterstained with hematoxylin.

### Measurement of serum FAPα antibody using ELISA

Blood samples were obtained via the tail vein from each mouse, and serum was collected. After precoating 96-well plates with FAPαc antigen (100 ng/well) and blocking with 3% bovine serum albumin for 1 h at room temperature, the serum samples were added in serial dilutions from 1:1000 to 1:128,000. The plates were then washed, and HRP-conjugated IgG (1:5000 dilution; Boster) was added for 1 h at room temperature. The plates were then washed, and the signals were developed using tetramethylbenzidine and hydrogen peroxide and were read at A_405_ using an Elx800 universal microplate reader (Bio-Tek).

### Detection of CD8+ T-cell function in the spleens of tumor bearing mice

Spleens were collected 31 days after tumor implantation. Freshly isolated splenocytes were incubated in ammonium chloride buffer to lyse the erythrocytes and then stimulated with 10 μg/ml FAPαc. IL-2 and Brefeldin A were added at concentrations of 300 U/ml and 1 μg/ml, respectively. After stimulation for 3 days, the cells were collected, stained with PE-CD8 and FITC-IFN-γ antibodies, and detected using flow cytometry.

### Statistical analyses

All values are reported as the mean ± SEM of three independent experiments unless otherwise specified. Comparisons between two groups were analyzed using two-tailed unpaired Student's *t* test, and one-way ANOVA followed by Bonferroni's test was used for multiple comparisons. These analyses were performed using Prism Software Version 5.0 (GraphPad Software Inc., La Jolla, CA). Values of *p* < 0.05 was considered statistically significant.
